# *In Situ* Study of Strain-Dependent Ion Conductivity of Stretchable Polyethylene Oxide Electrolyte

**DOI:** 10.1038/srep20128

**Published:** 2016-02-02

**Authors:** Taylor Kelly, Bahar Moradi Ghadi, Sean Berg, Haleh Ardebili

**Affiliations:** 1 Materials Science and Engineering Program, University of Houston, Houston, TX, 77204 USA; 2 Department of Mechanical Engineering, University of Houston, Houston, TX, 77204 USA

## Abstract

There is a strong need in developing stretchable batteries that can accommodate stretchable or irregularly shaped applications including medical implants, wearable devices and stretchable electronics. Stretchable solid polymer electrolytes are ideal candidates for creating fully stretchable lithium ion batteries mainly due to their mechanical and electrochemical stability, thin-film manufacturability and enhanced safety. However, the characteristics of ion conductivity of polymer electrolytes during tensile deformation are not well understood. Here, we investigate the effects of tensile strain on the ion conductivity of thin-film polyethylene oxide (PEO) through an *in situ* study. The results of this investigation demonstrate that both in-plane and through-plane ion conductivities of PEO undergo steady and linear growths with respect to the tensile strain. The coefficients of strain-dependent ion conductivity enhancement (CSDICE) for in-plane and through-plane conduction were found to be 28.5 and 27.2, respectively. Tensile stress-strain curves and polarization light microscopy (PLM) of the polymer electrolyte film reveal critical insights on the microstructural transformation of stretched PEO and the potential consequences on ionic conductivity.

Over the past decades, the conventional rigid batteries and supercapacitors have progressively evolved into smaller, thinner, and flexible devices^1^,^2^,^3^. More recently, a growing interest in developing stretchable energy storage devices has emerged^4^,^5^,^6^,^7^ to accommodate a wide range of stretchable applications including medical implants, textiles, and stretchable electronics. Most of the work for developing stretchable batteries has been focused on creating stretchable electrodes^4^,^5^,^6^ and stretchable serpentine electrical connectors for Li ion batteries^7^. In this study, we focus on stretchable solid polymer electrolytes (SPEs) for potential use in a fully stretchable battery.

There are several advantages to using SPEs over conventionally used liquid electrolytes, the most significant being enhanced safety due to the greater mechanical and chemical stability of SPEs^8^. However, SPEs cannot achieve comparable ion conductivities at room temperature. The polymer electrolytes that do exhibit higher ion conductivities typically lack sufficient mechanical stability – they are either in the form of a gel electrolyte (composed of solid and liquid phases) or consist of lower molecular weight polymers. Most common methods for achieving improved ion conductivity without compromising the mechanical integrity of the SPEs consist of introducing nanofillers^9^,^10^,^11^,^12^ into the polymer matrix or, alternatively, using polymer blends^8^,^9^,^13^,^14^,^15^. Poly(ethylene oxide) (PEO), one of the most extensively studied polymer electrolytes, is a semi-crystalline polymer with the ability to solvate large quantities of lithium salts^8^,^9^,^11^,^15^,^16^.

The ion conductivity of a polymer electrolyte is believed to arise mainly from the amorphous phase of the polymer, above the glass-transition temperature. The lithium ions are transported by the repeated thermally driven motion of the polymer chain segments, which creates new coordination sites that the ions use to migrate through the electrolyte^14^. The ion conductivity of the PEO based electrolytes is generally governed by the complex interplay of the ion transport along the helical chains in the PEO and its segmental motions^15^.

Golodnitsky and co-workers^17^,^18^,^19^,^20^,^21^,^22^,^23^,^24^,^25^ have studied the effect of stretching on the ion conductivity of hot-pressed PEO and found that stretching enhances the conductivity parallel to the stretching axis (in-plane) by a factor of 3–40^17^,^18^,^19^. The conductivity in the perpendicular direction (through-plane), however, has been observed to decrease and was attributed to the stiffening of the polymer chains, suppression in ion hopping between chains, and the alignment of PEO helices oriented in the force direction, leaving only a few helices oriented in the through-plane direction^17^. The measurements methods for in-plane and through-plane ion conductivities have also been discussed previously^26^,^27^.

In the present work, *in situ* impedance spectroscopy was performed on thin-film 600,000 Mw PEO/LiClO_4_ samples while the samples were subjected to tensile deformation (Fig. 1). Contrary to previous studies, the current work shows increased ion conductivity in both in-plane and through-plane conduction directions during the axial stretching of the polymer electrolyte. Specifically, the coefficients of strain-dependent ion conductivity enhancement (CSDICE) for in-plane and through-plane conduction were found to be 28.5 and 27.2, respectively. It is believed that this phenomenon stems from the microstructural changes occurring in the amorphous regions at the grain boundaries of the semi-crystalline polymer, namely, the unfolding and disentanglement of the polymer chains caused by mechanical deformation.

## Results and Discussions

**Figure 1:**
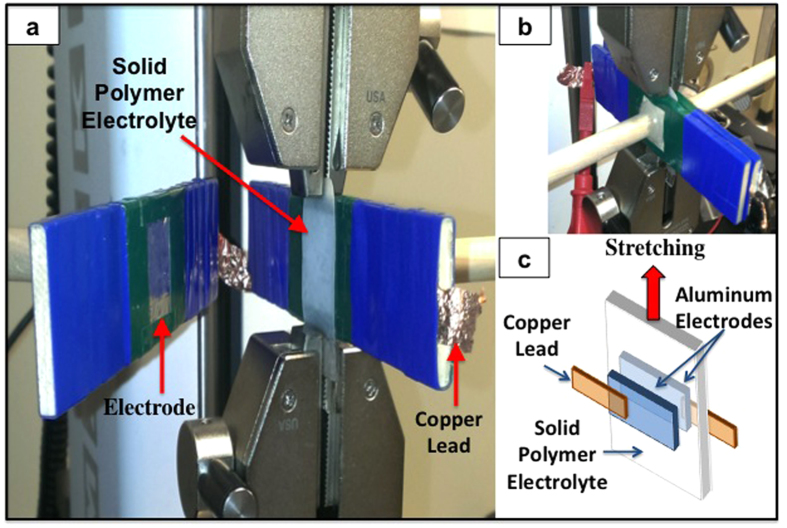
Experimental setup for *in situ* impedance spectroscopy during tensile stress-strain testing. (**a**) Electrode is in open position, (**b**) Closed electrodes, (**c**) Schematics of the experimental components and materials.

**Figure 2:**
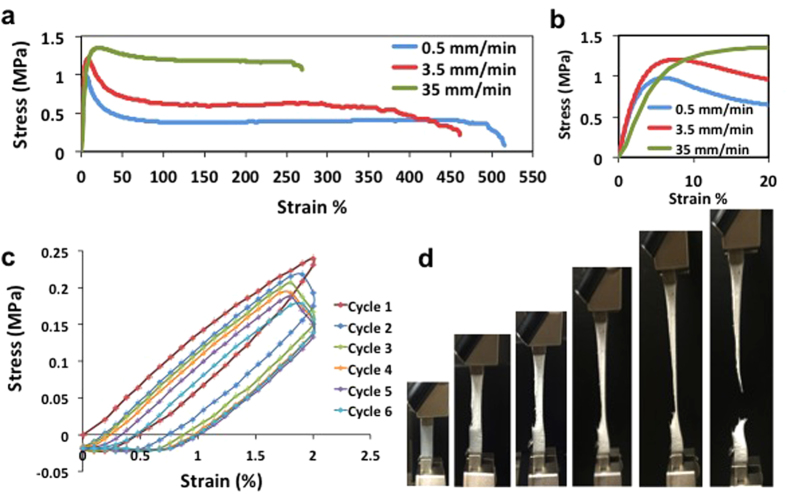
Tensile stress-strain behavior of solid PEO electrolyte film. (**a**) At three different strain rates, 0.5, 3.5 and 35 mm/min exhibiting up to 450% elongation (**b**) magnified curve below 20% strain, (**c**) stress-strain hysteresis effect, and (**c**) photo images of PEO sample subjected to tensile deformation.

Figure 1 shows the experimental setup used for the tensile displacement of the stretchable polymer electrolyte subjected to simultaneous-through-plane impedance spectroscopy. Figure 2a,b depict the representative stress-strain plots for solid PEO film at different strain rates (0.5, 3.5, and 35 mm/min) at 25 °C; additional stress-strain plots of the PEO are provided in the Supplementary Section (Figs S1, S2, S3, S4). The average yield strength, ultimate tensile strength, percentage elongation, and poison’s ratio are also reported in Table 1. If the behavior of a specimen is predominantly elastic in nature, the stresses are generally governed by forces carried within the amorphous and crystalline phase structures as well as viscous forces^28^. Semi-crystalline PEO exhibits a small elastic range, less than a 5% strain, at room temperature. This is expected as PEO is known to have a high degree of crystallinity (i.e. 60%) at room temperature, thus causing a relatively low ion conductivity at room conditions. For a specimen that exhibits tensile necking, the maximum stress indicates the onset of yielding, which is accompanied by a drop in stress in the engineering stress-strain curve. Void nucleation and growth occurs at yielding, which can be confirmed visually by the observed stress whitening. The degree of void nucleation and growth is dependent on whether or not particles are in contact with the void surfaces, which constrains the void^29^.

Observed in the tensile behavior of the PEO specimen, shown in Fig. 2c, is that the unload path differs from the load path, forming a hysteresis loop that could thermodynamically result in heat generation especially at high cycles^30^,^31^,^32^. Upon tensile reloading of the specimen in subsequent cycles, a noticeable drop in stress is observed at fixed strain. This is a feature of the viscoelasticity of the semicrystalline polymer and indicative of a type of mechanical damage^33^. Further cyclic loading shows a monotonic change in stress with cyclic loading which appears to approach an asymptotic value^34^.

**Figure 3:**
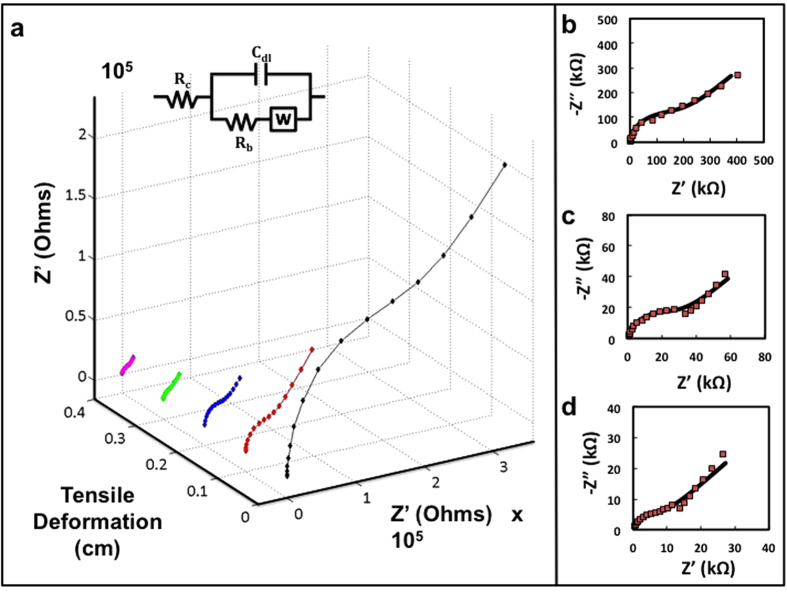
Impedance spectra of PEO in stretched and unstretched states. (**a**) Fitted nyquist plots for PEO at tensile deformation ranging from 0 to 0.4 cm; equivalent circuit used for fitting (inset), nyquist plots with equivalent circuit fit curves at (**b**) 0 cm, (**c**) 0.2 cm, and (**d**) 0.4 cm deformation.

Figure 3a,b show the through-plane nyquist plots for one sample of PEO stretched from 0 cm to 0.4 cm in 3D and 2D representations, respectively. The through-plane (z-axis) ion conductivity, *σ*_*z*_ of the electrolyte samples can be calculated using


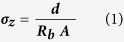


where *d* is the thickness of the electrolyte sample, *R*_*b*_ is the bulk resistance and *A* is the surface area of the electrolyte in contact with the electrode. The bulk resistance of the electrolyte can be estimated as the real impedance where the high frequency semi-circle and low frequency line intersect on the Nyquist plot. More accurately, electrolyte bulk resistance is determined from fitting of the equivalent electrochemical circuit to the Nyquist data (Fig. 3a, inset).

The through-plane ion conductivity is the typical conductivity used when characterizing an electrolyte for battery use because of the configuration of the cell where the electrolyte is sandwiched between the two electrodes. In this study, however, the in-plane (y-axis) conductivity was also determined in order to better understand the effect of axial strain on ion conduction through PEO. The in-plane ion conductivity, *σ*_*xy*_ can be calculated using Equation 2


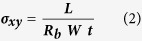


where *L* is the distance between the electrodes, *W* is the width of the sample and *t* is the sample thickness.

**Figure 4:**
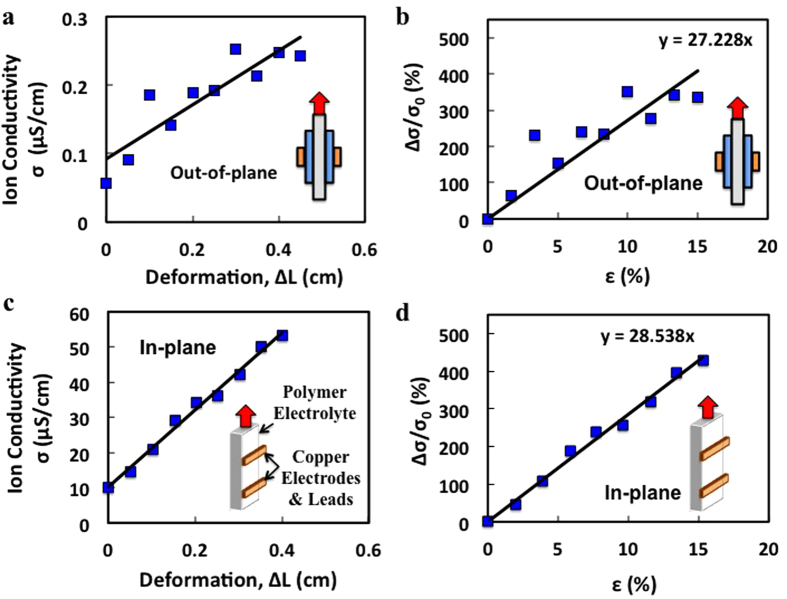
In-plane and through-plane ion conductivities of PEO electrolyte with respect to tensile deformation. (**a**) through-plane ion conductivity vs. tensile deformation of PEO/Li salt film, (**b**) Through -plane enhancement in ion conductivity vs. tensile strain, (**c**) In-plane ion conductivity vs. tensile deformation, (**d**) In-plane enhancement in ion conductivity vs. tensile strain.

Analysis of the data shows a clear effect of tensile deformation due to axial loading on ion conductivity in the PEO/Li salt polymer electrolyte, as seen in Fig. 4. The observed through-plane ion conductivity of unstretched PEO/LiClO_4_ sample, depicted in Fig. 4a and Table 2, is 5.6 × 10^−8^ S.cm^−1^ measured at about 18 °C. This value corresponds well to other published values for 600,000 Mw PEO^17^,^35^,^36^. The unstretched in-plane ion conductivity at the same temperature, in Fig. 4c, was observed to be 10^−6^, two orders of magnitude greater than that of the through-plane configuration. This phenomenon has been observed previously in several polymers and can be attributed to the order and orientation of polymer grains in the thin-film electrolyte^17^,^37^,^38^. At a slightly higher temperature of 25 ^o^C, both in-plane and through-plane ion conductivities increase as expected due to the thermally induced increase in both ion mobility and polymer segmental motion which can facilitate ion hopping between the coordinate sites on the polymer chains.

Figure 4a expresses the average ion conductivity versus tensile deformation distance and Fig. 4b depicts the average normalized ion conductivity of PEO with respect to strain for the through-plane configuration. Figure 4c,d show the same information for the in-plane configuration. Both conductivity directions show a similar and steady ion conductivity growth during axial deformation, achieving an approximate 4-fold increase in ion conductivity at a 15% strain.

Since the mechanical deformation and impedance spectroscopy of the polymer films were mainly performed in the ambient environment, further experiments were conducted to evaluate the potential effects of ambient moisture on ion conductivity during tensile deformation. Figure S6 shows the bulk resistance of an unstretched PEO film exposed to the ambient environment and Fig. S7 depicts the percent weight of absorbed moisture over time. For short periods of time, comparable to the testing times in this study (i.e. about 15 minutes), the results show that small amount of absorbed moisture has a negligible direct effect on the ion conductivity of unstretched PEO (Fig. S6a).

Figure S8 compares the percent change in ion conductivity with respect to the axial strain for 600,000 Mw PEO samples both inside and outside of the dry glove box. The results demonstrate that the ion conductivity of the PEO increases with strain in both ambient and dry environments. However, the ion conductivity of the polymer electrolyte appears to increase at a slower rate in the dry environment. This indicates that plasticization coupled with mechanical deformation of PEO electrolyte can lead to higher enhancement of ion conductivity. Tests on both plasticized and unplasticized PEO samples can offer insight into the potential electrochemical performance of PEO in a flexible or stretchable battery. Because most SPEs have relatively lower ion conductivities at room temperature, it is common practice to plasticize the solid polymer electrolyte inside a battery in order to enhance its ion conductivity and interface contact with the electrodes.

The increasing trend in ion conductivity with axial tensile strain infers that the structural changes induced in the polymer electrolyte results in altered and improved ion conduction. We hypothesize that the stretching and aligning of the amorphous polymer chains decreases the degree of tortuosity in the polymer, allowing for faster, and less obstructed ion transport through the polymer electrolyte.

**Figure 5:**
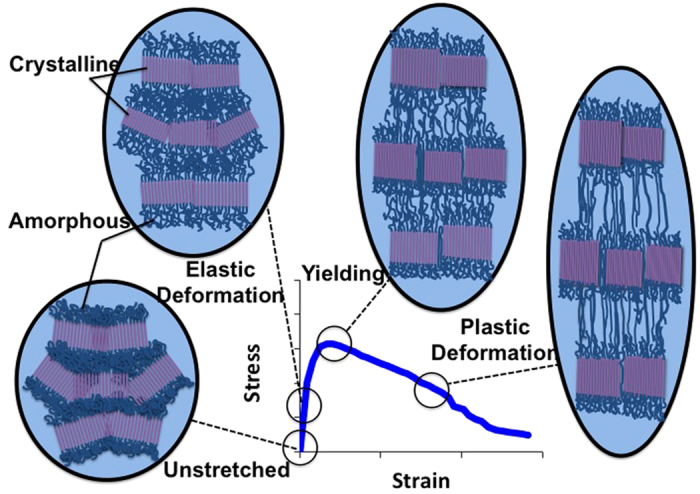
Depiction of semi-crystalline polymer microstructure at various stages of tensile deformation.

To better understand the mechanisms behind the ion conductivity enhancement in PEO, we shall delve deeper into the microstructural characteristics of the polymer. Semi-crystalline PEO consist of two phases, a crystalline phase and an amorphous phase as depicted in Fig. 5. In the crystalline phase, the ordered polymer chains align themselves in regions called crystallites. The amorphous phase of semi-crystalline PEO is generally present on the edges of the crystallites where the polymer chains are arranged in disordered, twisted and entangled conformations. The polymer chains in the amorphous regions predominantly tie one crystallite to another.

**Figure 6:**
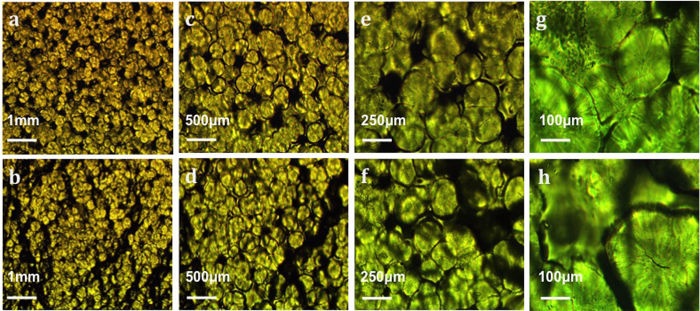
Polarization Light Microscopy (PLM) images of unstretched (top) and stretched (bottom) PEO. (**a,b**) 50x magnification, (**c,d**) 100x, (**e,f**) 200x, and (**g,h**) 500x magnifications.

To provide further insight into the effect of stretching on the molecular structure of the polymer electrolyte, polarization light microscopy (PLM) was performed on the PEO electrolyte samples. The PLM images in Fig. 6 are of the PEO/LiClO_4_ film in unstretched and stretched states at various magnifications. The images show a contrast of color where dark regions suggest amorphous phases and light regions indicate crystalline phases. The dark rings around the crystallites, visible at 100×–500× magnifications, are the entangled polymer chains tying one crystallite to its neighbor. The growth of dark regions, as the polymer transitions from the unstretched to stretched state, confirms a growth or extension of the amorphous regions as the chains stretch and disentangle.

The evolution of the microstructure of semicrystalline PEO is illustrated in Fig. 5. For small applied stress and strain, the polymer chains elongate elastically. The amorphous regions grow and transmit loading that is carried by the crystalline grains, and crystallite blocks slide and begin to orient into the direction of applied load^28^. The mechanical response of the crystalline region is elastic while the response of the amorphous region possess both elastic and viscoelastic behavior. Beyond the critical stress and the onset of yielding, favorably oriented crystalline grains subject to excessive shear begin to experience an increase in dislocations accompanied by transformation into fibrils^28^,^39^. With the increase in dislocations, void nucleation, growth and necking occur. As the process progresses the force carried dominantly by the crystallites shift to the amorphous regions until the chains are fully disentangled. These effects are believed to drive the nature of the stress-strain curves shown in Fig. 2.

The strength of the polymer mainly depends upon the degree of crystallinity, temperature, and density among others. In polyethylene oxide, the amorphous regions of the polymer are the most mechanically compliant part of the microstructure during applied tensile loading. The nature of inelastic deformation, or plasticity, in semi-crystalline polymers can be preceded by cavitation in the amorphous regions; however, the driving mechanisms depend on the strength of the crystallite grains and their susceptibility to shear deformation, void nucleation and growth^33^,^40^,^41^,^42^,^43^,^44^,^45^,^46^.

It is widely agreed upon that ion transport in SPEs is heavily dependent on the chain segmental motions of the polymer host. Increased motion creates more coordination sites that lithium ions can use to migrate from one electrode to the other. This is why it has long been believed that order and crystallinity adversely affects the ion conductivity of polymer electrolytes. Additionally, the conformation of amorphous polymer chains plays an important role in ion conductivity. The conformation and strength of polymer chains are dependent on several factors including the solvent used as well as the drying time^39^,^40^,^41^,^47^,^48^,^49^. Collapsed chain conformations are inefficient ion conductors because they trap and create a more tortuous migration path for lithium ions. Minimizing tortuosity by disentangling and opening up polymer chains via stretching can enhance ion conduction by increasing the chain’s ability to move, thus creating more coordination sites^13^,^50^.

Table 3 compares the slopes of the linear trends of the normalized conductivity versus strain data, which have been labeled as the coefficient of strain-dependent ion conduction enhancement (CSDICE). The CSDICE values for the through-plane and in-plane conduction are very similar, exhibiting values of 27.2 and 28.5, respectively. This suggests that ion conductivity growth in thin-film PEO with respect to the tensile strain is mainly an isotropic property, as growth in the through-plane and in-plane directions are found to be nearly the same. This further supports the theory that the conformations and entanglement of polymer chains unravel, which occurs in both the axial and lateral directions of the semi-crystalline polymer under tension.

Equation 3 is an empirical model for ion conductivity in stretched polyethylene oxide where *σ*_*0*_ is the ion conductivity of the sample at an unstretched state, *χ* refers to the CSDICE (presented in Table 3), and ε is the strain.


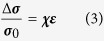


Based on the results obtained in this study, *χ* is an isotropic property, and therefore, Equation 3 can be valid for modeling both the through-plane and in-plane conductivities of PEO under axial strain.

Future studies should focus on the effect of material factors on strain-dependent ion conductivity, including chemically induced conformations of the polymer chains (i.e. polymer type, blends, etc.), filler types and dimensions, and salt chemistry, and provide further insight to the underlying phenomena through specialized experiments or numerical simulations. The effects of mechanical stress-strain cycling on the ion conductivity of polymer electrolyte should also be investigated in future work.

## Conclusions

In summary, *in situ* impedance spectroscopy during the tensile deformation of stretchable PEO/LiClO_4_ electrolyte film shows increase in both the through-plane and in-plane ion conductivities of the polymer electrolyte. This phenomenon is mainly attributed to the disentanglement of polymer chains during tensile deformation that, consequently, reduce the degree of tortuosity in the path of ion transport. The increase in ion conductivity of PEO follows a linear trend, and the slopes of the normalized conductivity vs. strain data termed as the CSDICE, for both the through-plane and in-plane experiments exhibit values of 27.2 and 28.5, respectively. The similarity of these values suggests that the CSDICE is an isotropic property of the polymer electrolyte, describing the physical interactions of polymer chains during stretching.

## Methods

### Polymer electrolyte fabrication

Thin-film solid polyethylene oxide (PEO) electrolyte was prepared in our laboratory using a solution-casting method. 2 g of PEO (Mw 600,000, Aldrich) and 0.3 g of LiClO_4_ (99.99%, Aldrich) were dispersed in anhydrous acetonitrile (99.9%, Sigma-Aldrich) and stirred at room temperature for 24 hours. The relatively high molecular weight (i.e. 600,000 Mw) PEO was selected because of its superior physical and electrochemical stability, especially in ambient conditions. The mixture of polymer, salt and solvent was then dried at 50 ^o^C under vacuum for another 24 hours creating a polymer film approximately 200–300 μm thick. All electrolyte films were stored in an argon-filled VAC glove box for at least 48 hours prior to use to ensure that any residual moisture had been removed.

### Thermogravimetric analysis (TGA)

Thermogravimetric analysis (TGA) desorption test was performed using TA Instruments TGA from 25 ^o^C to 200 ^o^C to verify the drying process of the PEO electrolyte films. The TGA of PEO electrolyte at various drying times is depicted in Fig. S5. TGA absorption test at room temperature (Fig. S7) was performed to quantify the moisture content in PEO absorbed over time when exposed to the ambient environment.

### *In situ* impedance spectroscopy setup

A layer of aluminum foil was attached to two wooden columns using a double-sided adhesive. Electrical insulating tape was used to mark off two identically sized squares and the exposed aluminum foil area acted as the electrodes. The two wooden columns, held in place using ring stands, were placed on either side of the sample such that the exposed aluminum surfaces were in contact with the polymer electrolyte, as seen in Fig. 1. Plastic pins were then placed across the wooden columns and used to minimize the contact resistance between the polymer and the electrodes. Finally, the copper leads were connected to the Autolab potentiostat for impedance spectroscopy. The nyquist plots from impedance spectroscopy of the polymer electrolyte film were obtained as the polymer was stretched in approximately 0.5 mm increments

### Mechanical and impedance test procedures

Mechanical testing of polymer electrolyte films was conducted outside of the glove box at ambient room conditions. *In situ* impedance spectroscopy tests were conducted both at ambient and dry conditions. Furthermore, the effect of variation in the ratio of the electrode to electrolyte areas during the tensile deformation of the polymer electrolyte was investigated. The established baseline measurements for room condition and area variation (Fig. S6) and micro-void formation (Fig. S7) were considered during the calculation of the ion conductivity of stretched polymer in order to precisely isolate the effect of tensile deformation on the ion conductivity of PEO/LiClO_4_ film.

The thin-film polymer electrolyte samples were loaded into a MARK-10 ESM301L motorized test stand and stretched at a strain rate of 3.5 mm/min. Through-plane and in-plane *in situ* complex impedance spectra of the solid polymer were obtained in the frequency range of 10 MHz–1 Hz using an Autolab multichannel potentiostat fitted with an FRA module for conducting electrochemical impedance spectroscopy (EIS) measurements. The effect of strain rate on tensile stress-strain curve was also investigated at strain rates of 0.5, 3.5, and 35 mm/min. Stress-strain cyclic tests were conducted on the PEO electrolyte film for 6 load/unload cycles to observe hysteresis effects.

## Additional Information

**How to cite this article**: Kelly, T. *et al.* *In Situ* Study of Strain-Dependent Ion Conductivity of Stretchable Polyethylene Oxide Electrolyte. *Sci. Rep.* **6**, 20128; doi: 10.1038/srep20128 (2016).

## Supplementary Material

Supplementary Information

## Figures and Tables

**Table 1:** Mechanical properties of thin-film 600,000 Mw PEO/LiClO_4_.

Material (Temperature, Strain Rate)		Yield Strength (MPa)	Tensile Strength (MPa)	% Elongation	Poisson’s Ratio
PEO (18 °C, 3.5 mm/min)		0.81	1.26	141	0.231
PEO (25 °C)	0.5 mm/min	0.64	0.99	509	0.241
	3.5 mm/min	0.66	1.02	457	0.236
	35 mm/min	0.91	1.54	278	0.238

**Table 2:** Ionic conductivity of thin-film 600,000 Mw PEO/LiClO_4_ in unstretched state.

	Ion Conductivity (S/cm)	
	Out-of-Plane	In-Plane
PEO (18 °C)	5.6 × 10^−8^	1.0 × 10^−6^
PEO (25 °C)	1.5 × 10^−7^	4.6 × 10^−5^

**Table 3:** Comparison of through-plane and in-plane CSDICE values.

Conductivity Direction	CSDICE ( 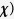
Through-plane	27.2
In-plane	28.5
