# Supplementary material for: In Situ Study of Strain-Dependent Ion Conductivity of Stretchable Polyethylene Oxide Electrolyte
Source: Sci Rep. 2016 Feb 2;6:20128. doi: 10.1038/srep20128 (PMC4735716; doi:10.1038/srep20128)
Supplement: Supplementary Information [file srep20128-s1.docx]

*In Situ* Study of Strain-Dependent Ion Conductivity of Stretchable Polyethylene Oxide Electrolyte

**Supplementary Information**

Taylor Kelly ^1^, Bahar Moradi Ghadi ^1^, Sean Berg ^1^, and Haleh Ardebili ^1,2*^

^1^ Materials Science and Engineering Program, University of Houston, Houston, TX, 77204 USA

^2^ Department of Mechanical Engineering, University of Houston, Houston, TX, 77204 USA

* Email: hardebili@uh.edu

**Figure S1** presents the results of the tensile strain-stress testing on PEO sample at 18^o^C. It also includes photo images of PEO polymer sample before and after a 4mm displacement. In all polymer samples, stress concentrations form in the areas surrounding the wedge clamps of the tensile tester. The white portions of the sample, seen in the image on the right, are cavitations, occurring during yielding due to axial strain. **Figures S2, S3, and S4** show the stress-strain plots for three samples at different strain rates at 25 ^o^C.


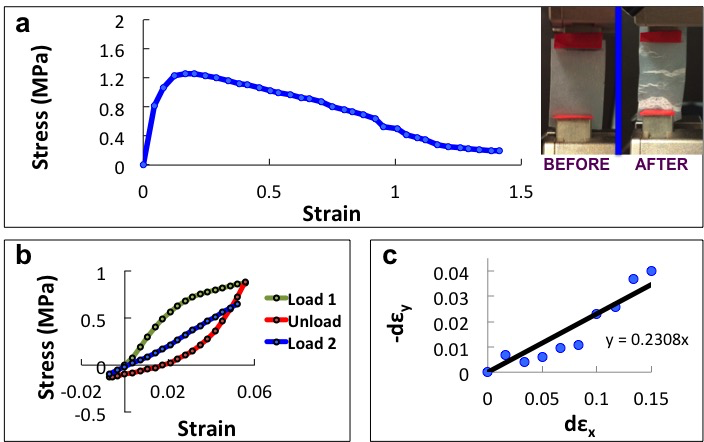


**Figure S1.** (a) Stress-strain curve of PEO/LiClO_4_ at 18^o^C; images of PEO electrolyte before and after stretching (inset), (b) hysteresis effect, (c) transverse vs. axial strain (Poisson’s ratio)


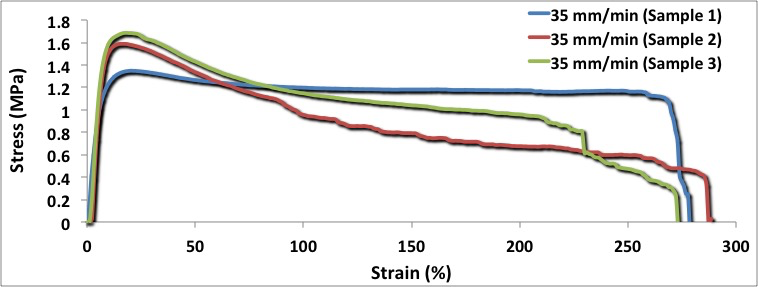


**Figure S2**. Tensile stress-strain plots of three samples of PEO/Li salt at 35 mm/min strain rate.


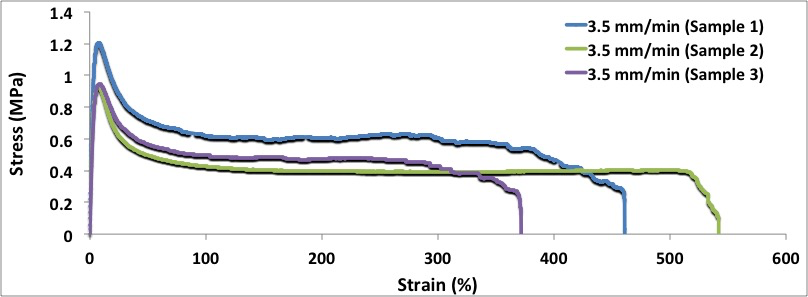


**Figure S3.** Tensile stress-strain plots of three samples of PEO/Li salt at 3.5 mm/min strain rate.


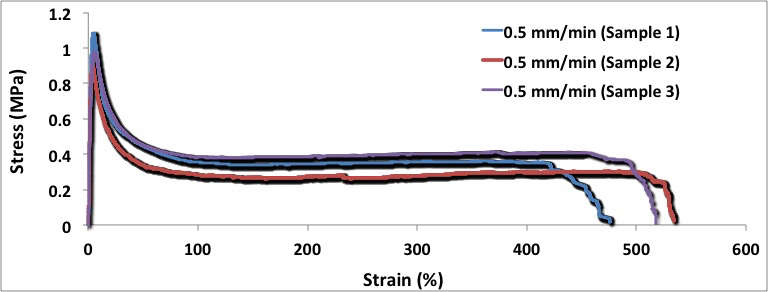


**Figure S4.** Tensile stress-strain plots of three samples of PEO/Li salt at 0.5 mm/min strain rate.


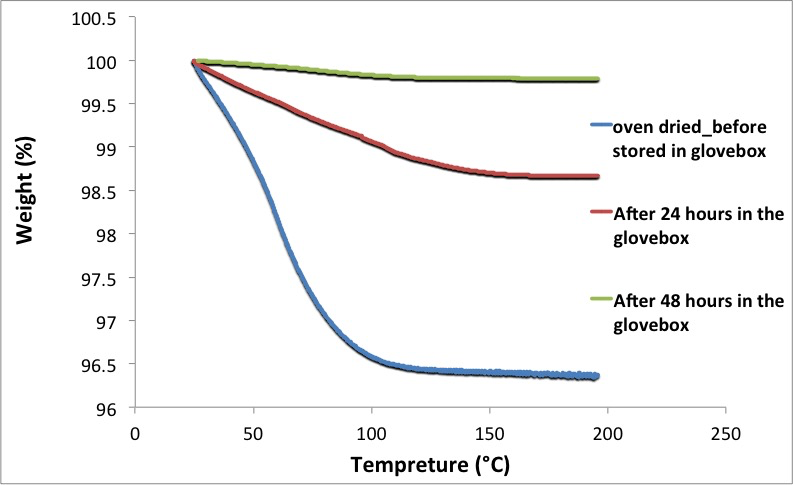


**Figure S5.** Thermogravimetric analysis (TGA) of PEO electrolyte at different drying times

**Figure S5** shows the thermogravimetric analysis (TGA) of PEO electrolyte film at different drying times. **Figure S6** shows the effects of moisture and electrode to electrolyte area on the bulk resistance of the PEO electrolyte. Because all ion conductivity measurements were taken outside of the glove box, the PEO samples were susceptible to moisture uptake. Moisture can act as a plasticizer, compromising the mechanical properties and decreasing the bulk resistance of the electrolyte. The bulk resistance of the PEO samples was measured over time in order to better understand the effect of moisture absorption on ion conductivity. These findings were used to correct all normalized conductivity vs. axial displacement data in order to isolate the effect of stretching on ion conductivity.

Additionally, the effect of electrode to electrolyte areas on the electrolyte conductivity was also investigated. Because of the nature of these tests, the area of the electrodes cannot be equivalent to the area of the exposed electrolyte surface. A relatively simple test measuring the bulk resistance of the electrolyte using variable sized electrodes while maintaining a constant area electrolyte was performed and the results show that the bulk resistance decreases, meaning ion conductivity increases, as the ratio of the electrode to electrolyte areas approaches 1. This data was used to further correct the ion conductivity vs. displacement results to better show the degree to which stretching and microstructural changes affect the polymer electrolyte’s ion conduction.


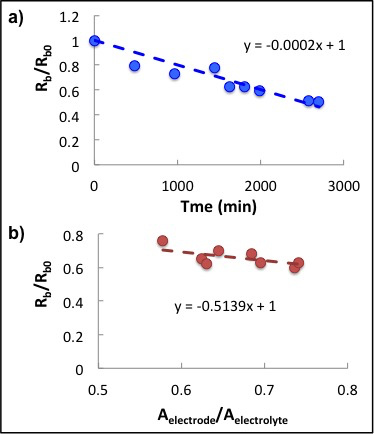


**Figure S6.** Effect of (a) moisture and (b) electrode to electrolyte area on bulk PEO resistivity

**Figure S7** provides a thermo-gravimetric analysis of (TGA) moisture content in both 600,000 Mw and 400,000 Mw PEO over time. **Figure S8** depicts the percent change of ion conductivity vs. axial strain for both 600,000 Mw and 400,000 Mw PEO in wet and dry environments. The CSDICE values obtained for the 400,000 Mw samples are less than those obtained for the 600,000 Mw samples.

**
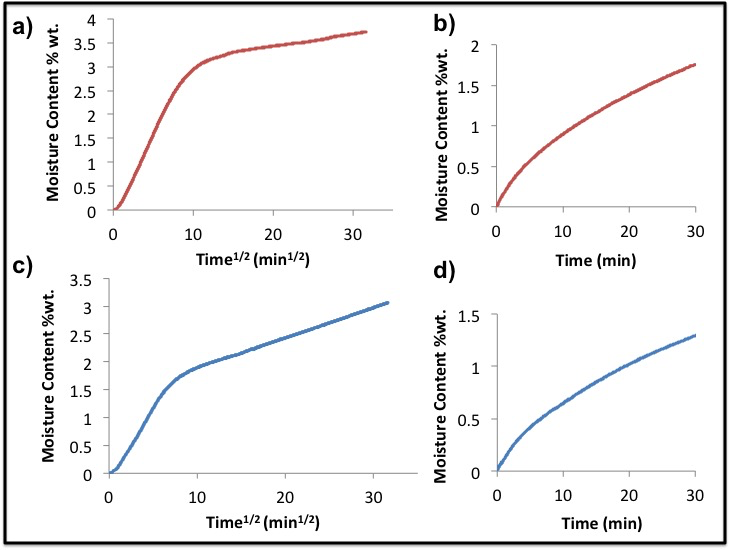
**

**Figure S7.** TGA at room temperature showing moisture absorption over time for a,b) 600,000 Mw PEO and c,d) 400,000 Mw PEO


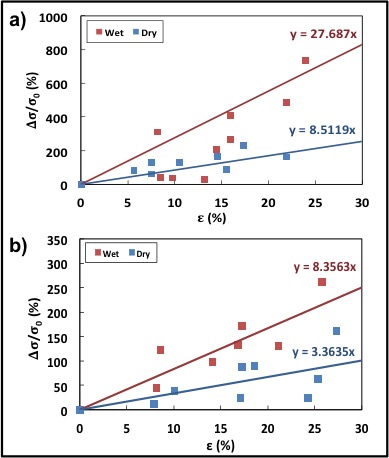


**Figure S8.** Percent change in ion conductivity vs. axial strain in wet and dry environments for

a) 600,000 Mw PEO and b) 400,000 Mw. PEO.

**Figure S9** depicts the effect of of microvoid formations compared to stretching on the ion conductivity of PEO. In the microvoid samples, sixteen voids were formed using the tip of a pin and placed in identical positions on three separate samples and another 3 samples were stretched to an arbitrary extent. The ion conductivity of all six samples were determined before and after deformation occurred. The microvoid samples (green) showed very little difference in ion conductivity before and after the microvoids were made. Stretching, on the other hand, showed repeatable results of increased ion conductivity in the PEO samples. This supports the hypothesis that microstructural changes due to stretching facilitate enhanced ion transport across the polymer electrolyte.


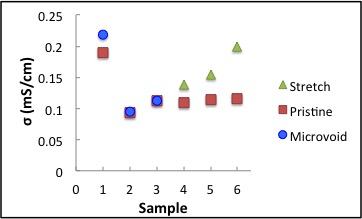


**Figure S9.** Comparison of the effects of microvoids and stretching on ion conductivity
